# Supplementary material for: Backward transfer entropy: Informational measure for detecting hidden Markov models and its interpretations in thermodynamics, gambling and causality
Source: Sci Rep. 2016 Nov 11;6:36831. doi: 10.1038/srep36831 (PMC5104982; doi:10.1038/srep36831)
Supplement: Supplementary Information [file srep36831-s1.pdf]

Supplementary Information: Backward transfer entropy: Informational measure for detecting hidden Markov models and its interpretations in thermodynamics, gambling and causality

Sosuke Ito[1,2]
[1]*Department of Physics, Tokyo Institute of Technology, Oh-okayama 2-12-1, Meguro-ku, Tokyo 152-8551, Japan*
[2]*FOM Institute AMOLF, Science Park 104, 1098 XG Amsterdam, The Netherlands*
(Dated: September 21, 2016)

Supplementary note 1 | Detailed derivation of the inequality (12) in the main text. To simplify the calculation, we use the notation $\rho(a) := p(A=a)$ and $\rho(a|b) := p(A=a|B=b)$ for any random variables A and B. We also use the notation $\rho_B(a|b) := p_B(A=a|B=b)$ for any random variables A and B. We define ensemble average as $\langle \cdots \rangle = \sum_{x_N^{(N)}, y_N^{(N)}} \rho(x_N^{(N)}, y_N^{(N)}) \cdots$. We here consider the following non-Markovian interacting dynamics,

$$\rho(x_N^{(N)}, y_N^{(N)}) := \rho(x_1, y_1) \prod_{k'=1}^{n} \rho(x_{k'+1}|x_{k'}, y_1)\rho(y_{k'+1}|y_{k'}, x_1) \prod_{k=n+1}^{N-1} \rho(x_{k+1}|x_k, y_{k-n})\rho(y_{k+1}|y_k, x_{k-n}), \tag{1}$$

with $n \geq 1$, and

$$\rho(x_N^{(N)}, y_N^{(N)}) := \rho(x_1, y_1) \prod_{k=1}^{N-1} \rho(x_{k+1}|x_k, y_k)\rho(y_{k+1}|y_k, x_k), \tag{2}$$

with $n = 0$.

To derive the inequality (12) in the main text, we calculate the difference between $I^n(X_N^{(N)} \to Y_N^{(N)}) - I^n(X^{\dagger}{}_N^{(N)} \to Y^{\dagger}{}_N^{(N)})$ and $-\Delta S_{\mathcal{X}B}$ in the case of $n \geq 1$ as follows;

$$\begin{aligned}
&\Delta S_{\mathcal{X}B} + I^n(X_N^{(N)} \to Y_N^{(N)}) - I^n(X^{\dagger}{}_N^{(N)} \to Y^{\dagger}{}_N^{(N)}) \\
=&\Delta S_{\mathcal{X}B} - \sum_{k=1}^{n} \left[T_{X^{\dagger}{}_1^{(1)} \to Y^{\dagger}{}_{k+1}^{(k+1)}} - T_{X_1^{(1)} \to Y_{k+1}^{(k+1)}} \right] - \sum_{k=n+1}^{N-1} \left[T_{X^{\dagger}{}_{k-n}^{(1)} \to Y^{\dagger}{}_{k+1}^{(k+1)}} - T_{X_{k-n}^{(1)} \to Y_{k+1}^{(k+1)}} \right] - I(X_N; Y_N) + I(X_1; Y_1) \\
=& \left\langle \ln \left[\frac{\rho(x_1|y_1)}{\rho(x_N|y_N)} \prod_{k'=1}^{n} \frac{\rho(x_{k'+1}|x_{k'}, y_1)}{\rho_{\mathrm{B}}(x_{k'}|x_{k'+1}, y_1)} \prod_{k=n+1}^{N-1} \frac{\rho(x_{k+1}|x_k, y_{k-n})}{\rho_{\mathrm{B}}(x_k|x_{k+1}, y_{k-n})} \right] \right\rangle + \left\langle \ln \left[\prod_{k'=1}^{n} \frac{\rho(y_{k'+1}|y_{k'}, x_1)}{\rho(y_{k'+1}|y_{k'}^{(k')})} \prod_{k=n+1}^{N-1} \frac{\rho(y_{k+1}|y_k, x_{k-n})}{\rho(y_{k+1}|y_k^{(k)})} \right] \right\rangle \\
&+ \left\langle \ln \prod_{k'=1}^{n} \frac{\rho(y_{N-k'}|y_N^{(k')})}{\rho(y_{N-k'}|x_N, y_{N-k'+1})} \prod_{k=n+1}^{N-1} \frac{\rho(y_{N-k}|y_N^{(k)})}{\rho(y_{N-k}|x_{N-k+n+1}, y_{N-k+1})} \right\rangle \\
=& \left\langle \ln \frac{\rho(x_1, y_1) \prod_{k'=1}^{n} \rho(x_{k'+1}|x_{k'}, y_1)\rho(y_{k'+1}|y_{k'}, x_1) \prod_{k=n+1}^{N-1} \rho(x_{k+1}|x_k, y_{k-n})\rho(y_{k+1}|y_k, x_{k-n})}{\rho(x_N, y_N) \prod_{k'=n+1}^{N-1} \rho_{\mathrm{B}}(x_{k'}|x_{k'+1}, y_{k'-n}) \prod_{m'=N-n}^{N-1} \rho(y_{m'}|y_{m'+1}, x_N) \prod_{k=1}^{n} \rho_{\mathrm{B}}(x_k|x_{k+1}, y_1) \prod_{m=1}^{N-n-1} \rho(y_m|y_{m+1}, x_{m+n+1})} \right\rangle \\
=& \sum_{x_N^{(N)}, y_N^{(N)}} \rho(x_N^{(N)}, y_N^{(N)}) \ln \frac{\rho(x_N^{(N)}, y_N^{(N)})}{\tilde{\rho}(x_N^{(N)}, y_N^{(N)})},
\end{aligned} \tag{3}$$

where we used $\rho(y_{k'+1}|y_{k'}^{(k')}, x_1) = \rho(y_{k'+1}|y_{k'}, x_1)$ with $k' \leq n$, $\rho(y_{k+1}|y_k^{(k)}, x_{k-n}) = \rho(y_{k+1}|y_k, x_{k-n})$ with $k \geq n+1$, $\rho(y_{N-k'}|x_N, y_N^{(k')}) = \rho(y_{N-k'}|x_N, y_{N-k'+1})$ with $k' \leq n$, $\rho(y_{N-k}|x_{N-k+n+1}, y_N^{(k)}) = \rho(y_{N-k}|x_{N-k+n+1}, y_{N-k+1})$ with $k \geq n+1$, and $\tilde{\rho}(x_N^{(N)}, y_N^{(N)})$ is defined as

$$\tilde{\rho}(x_N^{(N)}, y_N^{(N)}) := \rho(x_N, y_N) \prod_{k'=n+1}^{N-1} \rho_{\mathrm{B}}(x_{k'}|x_{k'+1}, y_{k'-n}) \prod_{m'=N-n}^{N-1} \rho(y_{m'}|y_{m'+1}, x_N) \prod_{k=1}^{n} \rho_{\mathrm{B}}(x_k|x_{k+1}, y_1) \prod_{m=1}^{N-n-1} \rho(y_m|y_{m+1}, x_{m+n+1}). \tag{4}$$

In the case of $n=0$, we have

$$
\begin{aligned}
&\Delta S_{\mathcal{X}B} + I^0(X_N^{(N)} \to Y_N^{(N)}) - I^0({X^\dagger}_N^{(N)} \to {Y^\dagger}_N^{(N)}) \\
=&\Delta S_{\mathcal{X}B} - \sum_{k=1}^{N-1} \left[T_{{X^\dagger}_k^{(1)} \to {Y^\dagger}_{k+1}^{(k+1)}} - T_{X_k^{(1)} \to Y_{k+1}^{(k+1)}} \right] - I(X_N;Y_N) + I(X_1;Y_1) \\
=&\left\langle \ln \left[\frac{\rho(x_1|y_1)}{\rho(x_N|y_N)} \prod_{k=1}^{N-1} \frac{\rho(x_{k+1}|x_k,y_k)}{\rho_{\mathrm{B}}(x_k|x_{k+1},y_k)} \right] \right\rangle + \left\langle \ln \left[\prod_{k=1}^{N-1} \frac{\rho(y_{k+1}|y_k,x_k)}{\rho(y_{k+1}|y_k^{(k)})} \right] \right\rangle + \left\langle \ln \prod_{k=1}^{N-1} \frac{\rho(y_{N-k}|y_N^{(k)})}{\rho(y_{N-k}|x_{N-k+1},y_{N-k+1})} \right\rangle \\
=&\left\langle \ln \frac{\rho(x_1,y_1)\prod_{k=1}^{N-1}\rho(x_{k+1}|x_k,y_k)\rho(y_{k+1}|y_k,x_k)}{\rho(x_N,y_N)\prod_{k=1}^{N-1}\rho_{\mathrm{B}}(x_k|x_{k+1},y_k)\prod_{k=1}^{N-1}\rho(y_k|y_{k+1},x_{k+1})} \right\rangle \\
=&\sum_{x_N^{(N)},y_N^{(N)}} \rho(x_N^{(N)},y_N^{(N)}) \ln \frac{\rho(x_N^{(N)},y_N^{(N)})}{\tilde{\rho}(x_N^{(N)},y_N^{(N)})},
\end{aligned} \tag{5}
$$

where we used $\rho(y_{k+1}|y_k^{(k)},x_k) = \rho(y_{k+1}|y_k,x_k)$, $\rho(y_N|x_{N-k+1},y_N^{(k)}) = \rho(y_{N-k}|x_{N-k+1},y_{N-k+1})$, and $\tilde{\rho}(x_N^{(N)},y_N^{(N)})$ is defined as

$$
\tilde{\rho}(x_N^{(N)},y_N^{(N)}) := \rho(x_N,y_N)\prod_{k=1}^{N-1}\rho_{\mathrm{B}}(x_k|x_{k+1},y_k)\prod_{m=1}^{N-1}\rho(y_m|y_{m+1},x_{m+1}). \tag{6}
$$

The function $\tilde{\rho}(x_N^{(N)},y_N^{(N)})$ is nonnegative, and satisfies the normalization of the probability;

$$
\begin{aligned}
&\sum_{x_N^{(N)},y_N^{(N)}} \tilde{\rho}(x_N^{(N)},y_N^{(N)}) \\
=&\sum_{x_N^{(N)},y_N^{(N)}} \rho(x_N,y_N) \prod_{k'=n+1}^{N-1} \rho_{\mathrm{B}}(x_{k'}|x_{k'+1},y_{k'-n}) \prod_{m'=N-n}^{N-1} \rho(y_{m'}|y_{m'+1},x_N) \prod_{k=1}^{n} \rho_{\mathrm{B}}(x_k|x_{k+1},y_1) \prod_{m=1}^{N-n-1} \rho(y_m|y_{m+1},x_{m+n+1}) \\
=&\sum_{x_N^{(N-n)},y_N^{(N)}} \rho(x_N,y_N) \prod_{k'=n+1}^{N-1} \rho_{\mathrm{B}}(x_{k'}|x_{k'+1},y_{k'-n}) \prod_{m'=N-n}^{N-1} \rho(y_{m'}|y_{m'+1},x_N) \prod_{m=1}^{N-n-1} \rho(y_m|y_{m+1},x_{m+n+1}) \\
=&\sum_{x_N^{(N-n-1)},y_N^{(N-1)}} \rho(x_N,y_N) \prod_{k'=n+2}^{N-1} \rho_{\mathrm{B}}(x_{k'}|x_{k'+1},y_{k'-n}) \prod_{m'=N-n}^{N-1} \rho(y_{m'}|y_{m'+1},x_N) \prod_{m=2}^{N-n-1} \rho(y_m|y_{m+1},x_{m+n+1}) \\
=&\cdots \\
=&\sum_{x_N^{(1)},y_N^{(n+1)}} \rho(x_N,y_N) \prod_{m'=N-n}^{N-1} \rho(y_{m'}|y_{m'+1},x_N) \\
=&\sum_{x_N^{(1)},y_N^{(1)}} \rho(x_N,y_N) \\
=&1,
\end{aligned} \tag{7}
$$

with $n \geq 1$, and

$$
\begin{aligned}
&\sum_{x_N^{(N)}, y_N^{(N)}} \tilde{\rho}(x_N^{(N)}, y_N^{(N)}) \\
&= \sum_{x_N^{(N)}, y_N^{(N)}} \rho(x_N, y_N) \prod_{k=1}^{N-1} \rho_{\mathrm{B}}(x_k | x_{k+1}, y_k) \prod_{m=1}^{N-1} \rho(y_m | y_{m+1}, x_{m+1}) \\
&= \sum_{x_N^{(N-1)}, y_N^{(N-1)}} \rho(x_N, y_N) \prod_{k=2}^{N-1} \rho_{\mathrm{B}}(x_k | x_{k+1}, y_k) \prod_{m=2}^{N-1} \rho(y_m | y_{m+1}, x_{m+1}) \\
&= \cdots \\
&= \sum_{x_N^{(1)}, y_N^{(1)}} \rho(x_N, y_N) \\
&= 1,
\end{aligned} \tag{8}
$$

with $n = 0$.

Thus Eqs. (3) and (5) are given by the Kullback-Libler divergence between $\rho(x_N^{(N)}, y_N^{(N)})$ and $\tilde{\rho}(x_N^{(N)}, y_N^{(N)})$. Because of the nonnegativity of the Kullback-Libler divergence [1], we have Eq. (12) in the main text

$$
-\Delta S_{\mathcal{X}B} \leq -\sum_{k=1}^{n} \left[T_{X\dagger_1^{(1)} \to Y\dagger_{k+1}^{(k+1)}} - T_{X_1^{(1)} \to Y_{k+1}^{(k+1)}} \right] - \sum_{k=n+1}^{N-1} \left[T_{X\dagger_{k-n}^{(1)} \to Y\dagger_{k+1}^{(k+1)}} - T_{X_{k-n}^{(1)} \to Y_{k+1}^{(k+1)}} \right] - I(X_N; Y_N) + I(X_1; Y_1), \tag{9}
$$

for $n \geq 1$, and

$$
-\Delta S_{\mathcal{X}B} \leq -\sum_{k=1}^{N-1} \left[T_{X\dagger_k^{(1)} \to Y\dagger_{k+1}^{(k+1)}} - T_{X_k^{(1)} \to Y_{k+1}^{(k+1)}} \right] - I(X_N; Y_N) + I(X_1; Y_1), \tag{10}
$$

for $n = 0$. The equality holds if and only if $\rho(x_N^{(N)}, y_N^{(N)}) = \tilde{\rho}(x_N^{(N)}, y_N^{(N)})$. In the case of $n = 0$, this condition is given by

$$
\rho(x_1, y_1) \prod_{k=1}^{N-1} \rho(x_{k+1} | x_k, y_k) \rho(y_{k+1} | y_k, x_k) = \rho(x_N, y_N) \prod_{k=1}^{N-1} \rho_{\mathrm{B}}(x_k | x_{k+1}, y_k) \prod_{m=1}^{N-1} \rho(y_m | y_{m+1}, x_{m+1}) \tag{11}
$$

$$
\rho(x_N, y_N) \prod_{k=1}^{N-1} \rho(x_k, y_k | y_{k+1}, x_{k+1}) = \rho(x_N, y_N) \prod_{k=1}^{N-1} \rho_{\mathrm{B}}(x_k | x_{k+1}, y_k) \prod_{m=1}^{N-1} \rho(y_m | y_{m+1}, x_{m+1}) \tag{12}
$$

$$
\prod_{k=1}^{N-1} \rho(x_k | y_k, y_{k+1}, x_{k+1}) = \prod_{k=1}^{N-1} \rho_{\mathrm{B}}(x_k | x_{k+1}, y_k), \tag{13}
$$

which implies the backward probability $\rho_{\mathrm{B}}(x_k | x_{k+1}, y_k)$ is equivalent to the original probability $\rho(x_k | y_k, y_{k+1}, x_{k+1})$. In a continuous limit, this fact implies the equality in the generalized second law (10) holds when the dynamics of $\mathcal{X}$ has a local reversibility, i.e., $\rho = \rho_B$.

Supplementary note 2 | Detailed calculation of Eqs. (17) and (18) in the main text.

We consider the following Markovian interacting dynamics

$$
\rho(x_N^{(N)}, y_N^{(N)}) = \rho(x_1, y_1) \prod_{k=1}^{N-1} \rho(x_{k+1} | x_k, y_k) \rho(y_{k+1} | x_k, y_k). \tag{14}
$$

From Eq. (14), we have $\rho(y_k | y_N^{(N-k)}, x_{k+1}) = \rho(y_k | y_{k+1}, x_{k+1})$ and $\rho(y_{k+1} | y_k^{(k)}, x_k) = \rho(y_{k+1} | y_k, x_k)$. We also have an identity $\rho(y_k | y_{k+1}) \rho(y_{k+1}) = \rho(y_{k+1} | y_k) \rho(y_k)$. Thus we can calculate the additivity Eq. (17) in the main text as

follows;

$$
\begin{aligned}
&I^0(X_N^{(N)} \to Y_N^{(N)}) - I^0(X^{\dagger}{}_N^{(N)} \to Y^{\dagger}{}_N^{(N)}) \\
&= \sum_{k=1}^{N-1} [T_{X_k^{(1)} \to Y_{k+1}^{(k+1)}} - T_{X^{\dagger}{}_k^{(1)} \to Y^{\dagger}{}_{k+1}^{(k+1)}}] + I(X_1; Y_1) - I(X_N; Y_N) \\
&= I(X_1; Y_1) + \sum_{k=1}^{N-1} I(X_k; Y_{k+1} | Y_k^{(k)}) - I(X_N; Y_N) - \sum_{k=1}^{N-1} I(X_{k+1}; Y_k | Y_N^{(N-k)}) \\
&= \left\langle \ln \left[\frac{\rho(y_N)\rho(y_1|x_1)}{\rho(y_1)\rho(y_N|x_N)} \prod_{k=1}^{N-1} \frac{\rho(y_k|y_N^{(N-k)})}{p(y_k|y_N^{(N-k)}, x_{k+1})} \frac{p(y_{k+1}|y_k^{(k)}, x_k)}{p(y_{k+1}|y_k^{(k)})} \right] \right\rangle \\
&= \left\langle \ln \left[\frac{\rho(y_1|x_1)}{\rho(y_N|x_N)} \prod_{k=1}^{N-1} \frac{\rho(y_k|y_{k+1})\rho(y_{k+1})}{p(y_k|y_{k+1}, x_{k+1})} \frac{\rho(y_{k+1}|y_k, x_k)}{\rho(y_{k+1}|y_k)\rho(y_k)} \right] \right\rangle \\
&= I(X_1; Y_1) + \sum_{k=1}^{N-1} I(X_k; Y_{k+1} | Y_k) - I(X_N; Y_N) - \sum_{k=1}^{N-1} I(X_{k+1}; Y_k | Y_{k+1}) \\
&= \sum_{k=1}^{N-1} [I(X_k; Y_{k+1} | Y_k) + I(X_k; Y_k) - I(X_{k+1}; Y_k | Y_{k+1}) - I(X_{k+1}; Y_{k+1})] \\
&= \sum_{k=1}^{N-1} \left[I^0(X_{k+1}^{(2)} \to Y_{k+1}^{(2)}) - I^0(X^{\dagger}{}_{N-k+1}^{(2)} \to Y^{\dagger}{}_{N-k+1}^{(2)}) \right]. \qquad (15)
\end{aligned}
$$

The difference between a tighter bound and DIF is calculated as follows;

$$
\begin{aligned}
&I^0(X_{k+1}^{(2)} \to Y_{k+1}^{(2)}) - I^0(X^{\dagger}{}_{N-k+1}^{(2)} \to Y^{\dagger}{}_{N-k+1}^{(2)}) + I_{\text{flow}}^k \\
&= I(X_k; \{Y_k, Y_{k+1}\}) - I(X_{k+1}; \{Y_k, Y_{k+1}\}) - I(X_k; Y_k) + I(X_{k+1}; Y_k) \\
&= I(X_k; Y_{k+1} | Y_k) - I(X_{k+1}; Y_{k+1} | Y_k) \\
&= \left\langle \ln \left[\frac{\rho(y_{k+1}|y_k, x_k)}{\rho(y_{k+1}|y_k, x_{k+1})} \right] \right\rangle \qquad (16)
\end{aligned}
$$

For the bipartite Markov jump process [2] or two dimensional Langevin dynamics without any correlation between thermal noises in $\mathcal{X}$ and $\mathcal{Y}$ [3], the ratio between two transition rates in $\mathcal{Y}$, i.e., $\langle \ln[\rho(y_{k+1}|y_k, x_k)/\rho(y_{k+1}|y_k, x_{k+1})] \rangle$ is up to order $O(\Delta t^2)$.

Supplementary note 3 | Comparison between a tighter bound in Eq. (16) and the result in [Ito, S., & Sagawa, T., Phys. Rev. Lett. 111, 180603 (2013)].

We compare a tighter bound in Eqs. (18) with our previous result in Ref. [3]. For the non-Markovian interacting dynamics

$$
\rho(x_N^{(N)}, y_N^{(N)}) := \rho(x_1, y_1) \prod_{k'=1}^{n} \rho(x_{k'+1}|x_{k'}, y_1)\rho(y_{k'+1}|y_{k'}, x_1) \prod_{k=n+1}^{N-1} \rho(x_{k+1}|x_k, y_{k-n})\rho(y_{k+1}|y_k, x_{k-n}), \qquad (17)
$$

with $n \geq 1$, the previous result in Ref. [3] gives the following bound of the entropy change in $\mathcal{X}$ and bath,

$$
\begin{aligned}
-\Delta S_{\mathcal{X}B} &\leq -\langle \Theta \rangle \\
&:= I(X_1; Y_1) + \sum_{k=1}^{n} T_{X_1^{(1)} \to Y_{k+1}^{(k+1)}} + \sum_{k=n+1}^{N-1} T_{X_{k-n}^{(1)} \to y_{k+1}^{(k+1)}} - I(X_N; Y_N^{(N)}) \\
&:= I^n(X_N^{(N)} \to Y_N^{(N)}) - I(X_N; Y_N) - \sum_{k=1}^{N-1} T_{X^{\dagger}{}_1^{(1)} \to Y^{\dagger}{}_{k+1}^{(k+1)}}
\end{aligned}
\qquad (18)
$$

where we used the identity $I(X_N; Y_N^{(N)}) = I(X_N; Y_N) + \sum_{k=1}^{N-1} T_{X^{\dagger}{}_1^{(1)} \to Y^{\dagger}{}_{k+1}^{(k+1)}}$. Here, $I(X_1; Y_1)$ corresponds to the initial correlation term I_{ini}, $\sum_{k=1}^{n} T_{X_1^{(1)} \to Y_{k+1}^{(k+1)}} + \sum_{k=n+1}^{N-1} T_{X_{k-n}^{(1)} \to y_{k+1}^{(k+1)}}$ corresponds the transfer entropy term $\sum_l I_{\text{tr}}^l$,

and $I(X_N;Y_N^{(N)})$ corresponds to the final correlation term $I_{\rm fin}$ in Ref. [3]. The difference between $-\langle\Theta\rangle$ and $I^n(X_N^{(N)} \to Y_N^{(N)}) - I^n(X^{\dagger(N)}_N \to Y^{\dagger(N)}_N)$ can be calculated as the difference of BTE,

$$-\langle\Theta\rangle - [I^n(X_N^{(N)} \to Y_N^{(N)}) - I^n(X^{\dagger(N)}_N \to Y^{\dagger(N)}_N)] = -\sum_{k=n+1}^{N-1} [T_{X^{\dagger(1)}_1 \to Y^{\dagger(k+1)}_{k+1}} - T_{X^{\dagger(1)}_{k-n} \to Y^{\dagger(k+1)}_{k+1}}]. \tag{19}$$

Due to the conditional Markov chain

$$\rho(x_N, x_{N-k+n+1}, y_{N-k}|y_N^{(k)}) = \rho(x_N|x_{N-k+n+1}, y_N^{(k)})\rho(x_{N-k+n+1}|y_{N-k}, y_N^{(k)})\rho(y_{N-k}|y_N^{(k)}), \tag{20}$$

we have the data processing inequality[1]

$$T_{X^{\dagger(1)}_1 \to Y^{\dagger(k+1)}_{k+1}} = I(X_N;Y_{N-k}|Y_N^{(k)}) \leq I(X_{N-k+n+1};Y_{N-k}|Y_N^{(k)}) = T_{X^{\dagger(1)}_{k-n} \to Y^{\dagger(k+1)}_{k+1}}. \tag{21}$$

Therefore, a tighter bound in inequality Eq. (16) is tighter than a bound in the previous result [3],

$$-\Delta S_{\mathcal{X}B} \leq I^n(X_N^{(N)} \to Y_N^{(N)}) - I^n(X^{\dagger(N)}_N \to Y^{\dagger(N)}_N) \leq -\langle\Theta\rangle. \tag{22}$$

Supplementary note 4 | Detailed derivation of the inequality (20) in the main text.

The set of side information x_k satisfies $x_{k-1} = \{s_1, \ldots, s_k\} \subset x_k$. Thus we have $\rho(y_{k+1}|y_k^{(k)}, x_k^{(k)}) = \rho(y_{k+1}|y_k^{(k)}, x_k)$ and $\rho(x_{k+1}|y_k^{(k)}, x_k^{(k)}) = \rho(x_{k+1}|y_k^{(k)}, x_k)$. The joint probability is given by

$$\rho(x_N^{(N)}, y_N^{(N)}) = \rho(x_1, y_1) \prod_{k=1}^{N-1} \rho(x_{k+1}|y_k^{(k)}, x_k)\rho(y_{k+1}|y_k^{(k)}, x_k). \tag{23}$$

Thus, we can calculate as follows;

$$\begin{aligned}
& - G + \sum_{k=1}^{N} \langle \ln o_k \rangle - S(Y_N^{(N)}) + I^0(X_N^{(N)} \to Y_N^{(N)}) \\
&= -\langle \ln f_1(y_1|x_1)\rangle - \sum_{k=1}^{N-1} \langle \ln f_{k+1}(y_{k+1}|y_k^{(k)}, x_k)\rangle + \langle \ln \rho(y_N^{(N)})\rangle + \left\langle \ln\left[\frac{\rho(x_1,y_1)}{\rho(x_1)\rho(y_1)}\right]\right\rangle + \left\langle \ln\left[\prod_{k=1}^{N-1} \frac{\rho(y_{k+1}|y_k^{(k)}, x_k)}{\rho(y_{k+1}|y_k^{(k)})}\right]\right\rangle \\
&= \left\langle \ln\left[\frac{\rho(x_1,y_1)}{f_1(y_1|x_1)\rho(x_1)} \prod_{k=1}^{N-1} \frac{\rho(y_{k+1}|y_k^{(k)}, x_k)}{f_{k+1}(y_{k+1}|y_k^{(k)}, x_k)}\right] + \ln\frac{\rho(y_N^{(N)})}{\rho(y_N^{(N)})}\right\rangle \\
&= \left\langle \ln\left[\frac{\rho(x_1,y_1)}{f_1(y_1|x_1)\rho(x_1)} \prod_{k=1}^{N-1} \frac{\rho(x_{k+1}|y_k^{(k)}, x_k)\rho(y_{k+1}|y_k^{(k)}, x_k)}{\rho(x_{k+1}|y_k^{(k)}, x_k)f_{k+1}(y_{k+1}|y_k^{(k)}, x_k)}\right]\right\rangle \\
&= \sum_{x_N^{(N)}, y_N^{(N)}} \rho(x_N^{(N)}, y_N^{(N)}) \ln\frac{\rho(x_N^{(N)}, y_N^{(N)})}{\pi(x_N^{(N)}, y_N^{(N)})},
\end{aligned} \tag{24}$$

where $\pi(x_N^{(N)}, y_N^{(N)})$ is defined as

$$\pi(x_N^{(N)}, y_N^{(N)}) := f_1(y_1|x_1)\rho(x_1) \prod_{k=1}^{N-1} \rho(x_{k+1}|y_k^{(k)}, x_k)f_{k+1}(y_{k+1}|y_k^{(k)}, x_k). \tag{25}$$

The function $\pi(x_N^{(N)}, y_N^{(N)})$ satisfies the normalization of the probability,

$$\begin{aligned}
\sum_{x_N^{(N)}, y_N^{(N)}} \pi(x_N^{(N)}, y_N^{(N)}) &= \sum_{x_N^{(N)}, y_N^{(N)}} f_1(y_1|x_1)\rho(x_1) \prod_{k=1}^{N-1} f_{k+1}(y_{k+1}|y_k^{(k)}, x_k)\rho(x_{k+1}|y_k^{(k)}, x_k) \\
&= \sum_{x_{N-1}^{(N-1)}, y_{N-1}^{(N-1)}} f_1(y_1|x_1)\rho(x_1) \prod_{k=1}^{N-2} f_{k+1}(y_{k+1}|y_k^{(k)}, x_k)\rho(x_{k+1}|y_k^{(k)}, x_k) \\
&= \cdots \\
&= \sum_{x_1, y_1} f_1(y_1|x_1)\rho(x_1) \\
&= \sum_{x_1} \rho(x_1) \\
&= 1, \qquad (26)
\end{aligned}$$

where we used $\sum_{y_1} f_1(y_1|x_1) = 1$ and $\sum_{y_{k+1}} f_{k+1}(y_{k+1}|y_k^{(k)}, x_k) = 1$.

Thus Eq. (24) is the Kullback-Libler divergence between $p(x_N^{(N)}, y_N^{(N)})$ and $\pi(x_N^{(N)}, y_N^{(N)})$. Because of the nonnegativity of the Kullback-Libler divergence [1], we have Eq. (20) in the main text

$$-G + \sum_{k=1}^{N} \langle \ln o_k \rangle - S(Y_N^{(N)}) + I^0(X_N^{(N)} \to Y_N^{(N)}) \geq 0. \qquad (27)$$

The equality holds if and only if $p(x_N^{(N)}, y_N^{(N)}) = \pi(x_N^{(N)}, y_N^{(N)})$. This condition is given by

$$\begin{aligned}
\rho(x_1, y_1) \prod_{k=1}^{N-1} \rho(x_{k+1}|y_k^{(k)}, x_k)\rho(y_{k+1}|y_k^{(k)}, x_k) &= f_1(y_1|x_1)\rho(x_1) \prod_{k=1}^{N-1} \rho(x_{k+1}|y_k^{(k)}, x_k) f_{k+1}(y_{k+1}|y_k^{(k)}, x_k) \\
\rho(y_1|x_1) \prod_{k=1}^{N-1} \rho(y_{k+1}|y_k^{(k)}, x_k) &= f_1(y_1|x_1) \prod_{k=1}^{N-1} f_{k+1}(y_{k+1}|y_k^{(k)}, x_k), \qquad (28)
\end{aligned}$$

which implies the bet fraction f_k is equivalent to the original probability ρ, i.e., the proportional betting is optimal.

[1] Cover, T. M., & Thomas, J. A., "*Elements of Information Theory*" (John Wiley and Sons, New York, 1991).

[2] Hartich, D., Barato, A. C., & Seifert, U., Stochastic thermodynamics of bipartite systems: transfer entropy inequalities and a Maxwell ' s demon interpretation. J. Stat. Mech. (2014) P02016.; In Appendix C, authors have shown that the ratio $\langle \ln[\rho(y_{k+1}|y_k, x_k)/\rho(y_{k+1}|y_k, x_{k+1})] \rangle$ is up to $O(\Delta t^2)$ for the bipartite Markov jump system [Eq. (C.11)].

[3] Ito, S., & Sagawa, T., Information thermodynamics on causal networks. Phys. Rev. Lett. **111**, 180603 (2013); In supplementary information C, Eqs. (15) and (16) can be interpreted as the fact that the ratio $\langle \ln[\rho(y_{k+1}|y_k, x_k)/\rho(y_{k+1}|y_k, x_{k+1})] \rangle$ is up to $O(\Delta t^2)$ for two dimensional Langevin dynamics without any correlation between thermal noises in $\mathcal{X}$ and $\mathcal{Y}$.
